# Prevalence of lead toxicity in adolescents in Kuwait

**DOI:** 10.1186/s12889-021-11210-z

**Published:** 2021-06-22

**Authors:** Reem Jallad, Muddanna S. Rao, Abdur Rahman

**Affiliations:** 1 Department of Food Science and Nutrition, College of Life Sciences, Kuwait University, Box 5969, 13060 Safat, Kuwait; 2 Department of Anatomy, Faculty of Medicine, Kuwait University, Box: 24923, 13110 Safat, Kuwait

**Keywords:** Lead toxicity, Adolescents, Kuwait, Prevalence, Cross-sectional

## Abstract

**Background:**

Elevated blood lead level (EBLL) is a public health problem in both developing and industrialized countries. Being a petrochemical-based economy, lead (Pb) levels are expected to be high in Kuwait, but systematic data on population exposure are lacking. This study aimed at determining the prevalence of EBLL in adolescents in Kuwait.

**Methods:**

Adolescents (*N* = 1385; age range 11–16 years) were cross-sectionally selected from public middle schools from all Governorates of Kuwait, utilizing multistage cluster random sampling. Pb in whole blood was analyzed by inductively coupled plasma mass spectrometry. Distribution of blood Pb levels (BLL) among Governorates and sexes were compared by non-parametric tests and the prevalence of EBLL (defined as BLL above the CDC reference level of ≥5 μg/dL) was estimated by χ^2^ test. Binary logistic regression was used for assessing the association between EBLL and Governorate.

**Results:**

Median (IQR) BLL was 5.1(3.6–7.1) μg/dL [4.9 (3.8–6.5) μg/dL in males and 5.4 (3.3–7.6) μg/dL in females; *p* = 0.001]. In the overall sample, 51% had BLL ≥5 μg/dL; 13% had ≥10 μg/dL and 3% > 20 μg/dL. Prevalence of EBLL was 47% in males and 56% in females (*p* < 0.001). EBLLs were clustered in Al-Asima, Al-Ahmadi (in both sexes); Al-Jahra (in males) and Mubarak Al-Kabeer (in females) Governorates.

**Conclusions:**

EBLL is a significant public health problem in adolescents in Kuwait. Urgent public health intervention is required in areas with EBLL, and the sources of exposure need to be identified for prevention.

**Supplementary Information:**

The online version contains supplementary material available at 10.1186/s12889-021-11210-z.

## Background

Lead (Pb) is a toxic metal with serious and long-lasting health consequences, particularly in children [1]. Due to its high malleability and resistance to corrosion, Pb has been used in various industries like plumbing, mining and metals recycling, in paint, pipes, batteries, cans, and cable covers, and in gasoline as an anti-knocking agent [2]. Due to its widespread use in many industries, Pb is present in our environment and humans are still exposed to it through food, water and air [3]. Once Pb enters the body, it is distributed in blood, soft tissues (e.g. liver, kidney, brain), and bones. With time and continuous exposure, Pb accumulates in bones where it resides for years. This stored Pb becomes a source of internal exposure, as it is released into circulation in conditions that favor calcium mobilization such as abnormal calcium homeostasis or pregnancy [4, 5].

Pb is a multisystem toxicant associated with neurological, nephrological, cardiac, gastrointestinal and hematological manifestations [3]. Based on accumulating evidence regarding the neurotoxic effects of Pb in children, the reference blood lead level (BLL) at which intervention needs to be initiated has progressively decreased from 60 μg/dL in the 1960s to 10 μg/dL in 1991 [6]. In 2012, the CDC stated that no level of Pb exposure in children is safe and established a new cutoff of 5 μg/dL as a reference level [7]. The debate continues, and some researchers in the field of Pb toxicity are suggesting lowering this level to < 3.5 μg/dL [8].

Kuwait’s economy is heavily based on its petrochemical industry. Large oil refineries and other petrochemical installations are located in the country [9]. In addition, it is a country with one of the heaviest traffic burdens [10]. As such, Pb pollution in the atmosphere is expected to be high. This atmospheric Pb can get into the food chain and become a source of oral ingestion through food and water [11]. The desert climate of Kuwait facilitates the spread of suspended Pb particles in the air and soil into residential areas [12, 13]. Air samples collected from several urban areas of Kuwait showed high Pb content in the 10-μm size particulate matter (PM_10_). The PM_10_ Pb content on average was 2.4 times higher than corresponding background Pb levels in the soil [14]. High levels of Pb in various foods, both locally grown and imported, have been reported [15–17]. Drinking water was also reported to be a source of Pb in older reports [18]; however, recent reports do not support water to be a major source of Pb contamination [19]. Smoking tobacco, which is a source of Pb exposure, is also common in Kuwait [20].

Despite these facts, systematic data on the population Pb exposure from Kuwait is scarce apart from a few old sporadic studies [21, 22]. Recent data on population Pb exposure are of interest to re-evaluate the global prevalence of Pb exposure after the revision of the reference level by the CDC in 2012. The majority of the data post-2012 are from occupationally exposed groups or from those living near a source of Pb. Data on general population exposure, particularly on adolescents, are meager. The adolescent population in Kuwait comprises approximately 1/4th of the total population. The objective of this study was to evaluate the prevalence of elevated blood lead levels (EBLL), defined as the BLL above the CDC reference level (5 μg/dL), in a representative sample of adolescents selected from public middle schools from all Governorates of Kuwait. Thus, the findings of this study would be both locally and globally important as it will fill the gap in the literature on Pb exposure among the adolescent population.

## Methods

### Study design and protocol

**Fig. 1:**
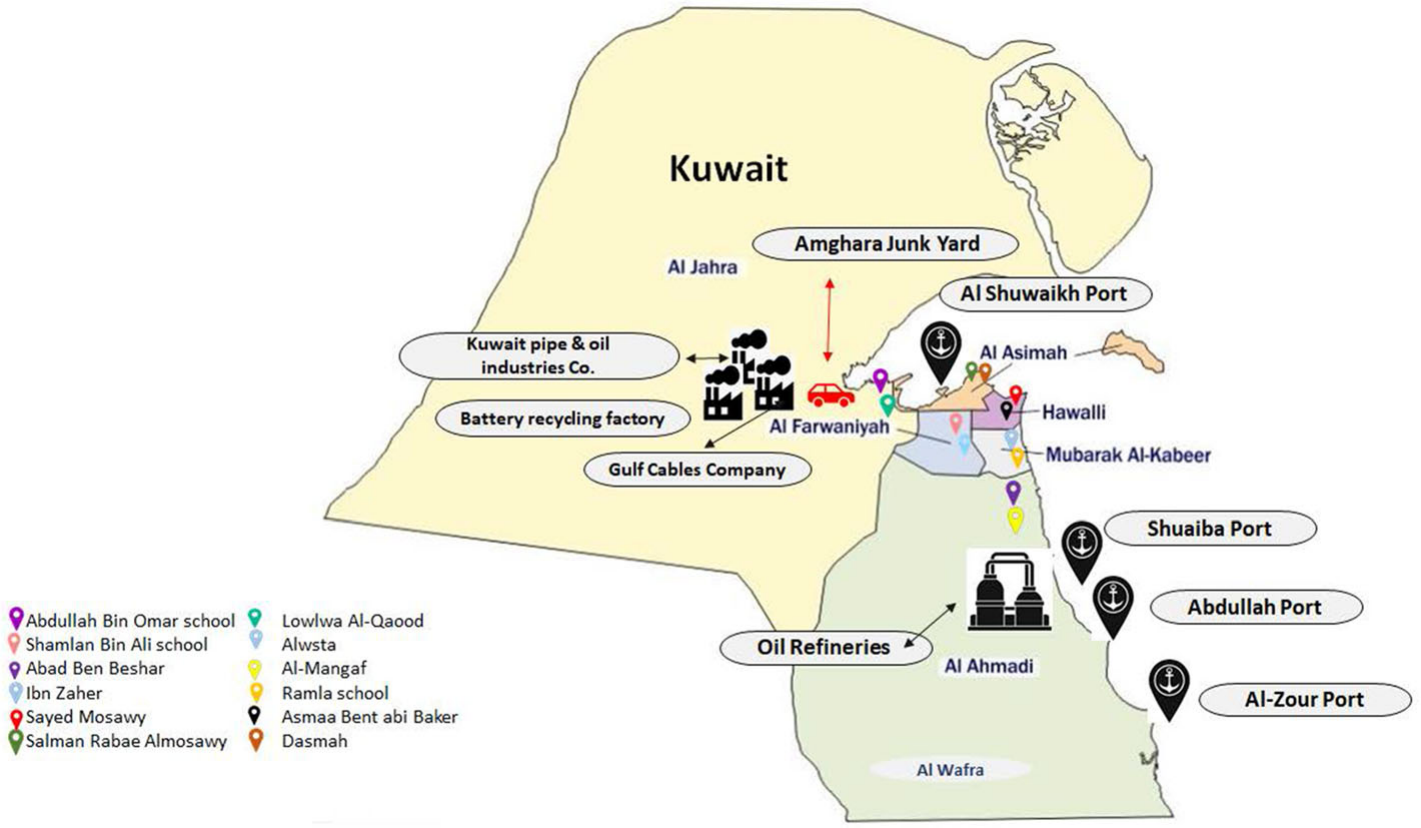
Kuwait map showing locations of selected schools and potential sources of exposure. The basic outline of the map was taken from the openly available sources from the internet, and final figure with the names of schools and potential sources of Pb exposure in each Governorate was prepared by the authors

This was a cross-sectional study conducted in public schools in Kuwait. Kuwait is divided into six Governorates: Al-Asima, Hawalli, Al-Ahmadi, Farwaniya, Al-Jahra and Mubarak Al-Kabeer (Fig. 1). There are separate male and female schools, and school enrollment reaches almost 100% for both males and females in Kuwait. The study population was students from middle schools (grades 6, 7 and 8) in the age range of 11 to 16 years. In order to select a representative sample of male and female students, a multistage cluster random sampling method with probability proportionate to size was used. The sample allocation in each Governorate was based on the relative size of that Governorate as judged by the total number of students in the selected age range (obtained from the General Population Census). From the list of all public schools, two schools (one male and one female) from each Governorate were selected. Schools with a larger student population had a higher probability of being selected compared to schools with a lower number of students. In total, students were selected from 12 schools. Details of the sampling, acquisition of data, and the study subject characteristics were reported in detail previously [23]. The study was approved by the Ethics Committee at the Ministry of Health, Kuwait (No: 2015/248) as well as the Ethics Committee of the Health Sciences Centre, Kuwait University (No: DR/EC/2338). The study was conducted in accordance with Declaration of Helsinki ethical principles for medical research involving human subjects. A written informed consent from the parents and verbal ascent of each participant was obtained.

### Demographic information and anthropometric measurements

Information on demographic variables were collected from parents through a self-administrative questionnaire and from students through face-to-face interviews using a structured questionnaire (Supplementary material, Data Acquisition Tool). Height and weight of each student was measured using a digital weight and height scale (Detecto, Webb City, MO, USA), with the students standing erect without shoes and wearing light clothes.

### Blood collection and Pb analyses

Blood samples were collected over a three-month period from February–April 2016. After obtaining written informed consent of the parents and verbal ascent of the students, 5 mL of venous blood was collected from each subject by a highly-trained pediatric nurse. Complete blood counts (CBC), including hemoglobin (Hb) concentration, were measured in a tertiary care hospital. For Pb analysis, whole blood samples (0.5 mL) were digested in a 5 mL perchloric acid/nitric acid (1:5) solution. After complete digestion of the samples, the perchloric acid/nitric acid solution was evaporated and the residue dissolved in 5 mL of 1% nitric acid. Double-deionized water was used in all sample preparation steps to avoid contamination. Pb was analyzed with Inductively Coupled Plasma Mass Spectrometry (ICP-MS). For a quality-control check, lyophilized whole blood samples of known mineral content (Clin-Check- Control, Cat. # 884042; Recipe, Munich, Germany) were included in the analyses.

### Statistics

Data was analyzed with SPSS version 26. Body mass index (BMI) was calculated as weight (Kg) divided by height squared (m^2^), and weight status was classified based on the BMI-for-age z-scores (zBMI) calculated using WHO growth charts. Students were classified as non-anemic or anemic based on the WHO age- and sex-specific cutoff points of Hb concentration. As Pb levels are not normally distributed, data are presented as median and interquartile range (IQR). Distribution of BLL among various demographic groups were compared with Mann-Whitney or Kruskall-Wallis tests as appropriate. Frequencies of students in various levels of BLL among various demographic groups were compared by a Chi-square (χ^2^) test. BLL of ≥5 μg/dL was defined as elevated blood lead level (EBLL). The odds ratio (OR) with 95% confidence interval (CI) of having EBLL in different Governorates and other demographic variables were calculated using a binary logistic regression in which BLLs were categorized as < 5μg/dL or ≥ 5 μg/dL (EBLL). Factors with *p* < 0.05 were deemed to be statistically significant.

## Results

**Fig. 2:**
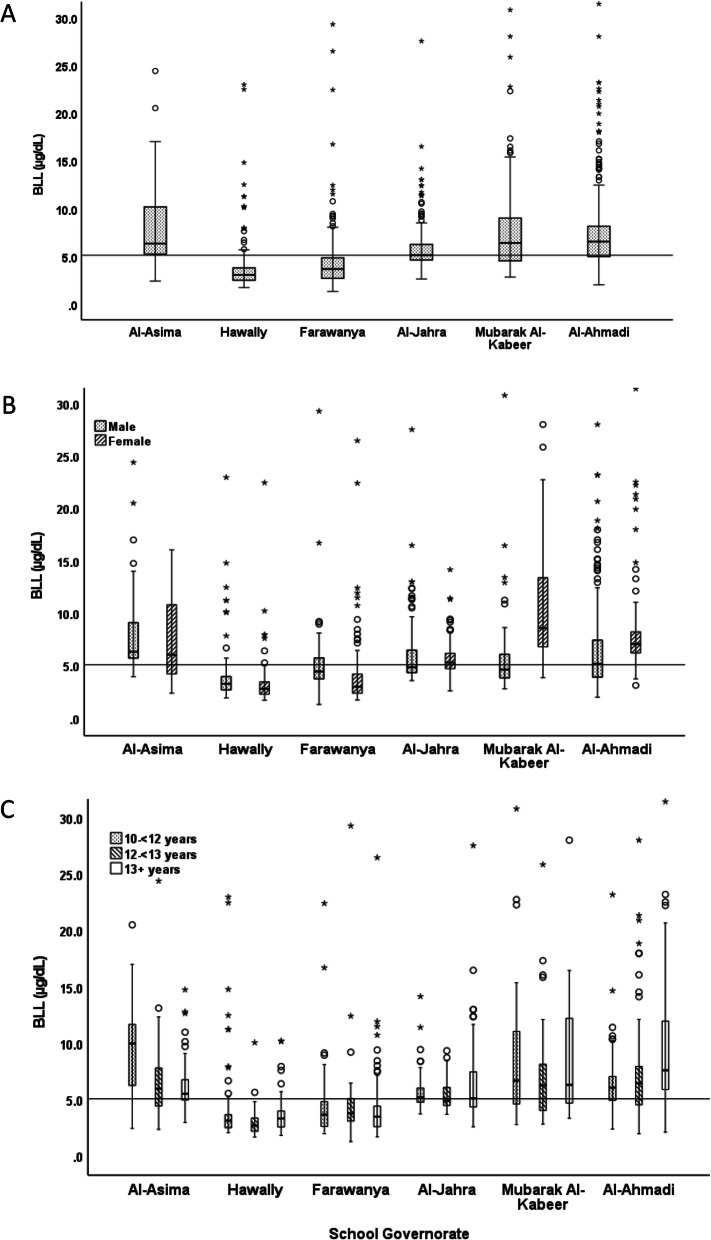
Boxplot showing distribution of Pb levels (μg/dL) stratified by Governorates (**A**), Governorates and sex (**B**), Governorates and age categories (**C**). Boxes show Pb distribution as median and interquartile range. Extremes values are shown above each box. For presentation, the maximum at Y-axis was set at 30 μg/dL. A total of 16 cases had blood Pb levels > 30 μg/dL. These are distributed as follows: Male; 1 in Hawally, 1 in Mubarak Al-Kabeer and 3 in Al-Ahmadi. Female; 1 in Al-Asima, 1 in Farwaniya, 1 in Al-Ahmadi and 8 in Mubarak Al-Kabeer. Statistics: A; Median test *p* < 0.001; B; Governorate, χ^2^ = 65.5, p < 0.001; sex, χ^2^ = 15.3, p < 0.001, Governorate*sex, χ^2^ = 94.5, p < 0.001; C; Governorate, χ^2^ = 149.2, p < 0.001; age, χ^2^ = 15.6, p < 0.001, Governorate*age, χ^2^ = 49.6, p < 0.001. Horizontal line represents the overall median. BLL: Blood Pb level

A total of 1583 households were recruited through schools, of which 1422 returned the consent forms with written approval. Blood samples were collected from 1416 students (6 students declined to give a blood sample). Pb data were available for 1385 adolescents, of which 673 (48.6%) were males. The mean (SD) age was 12.48 (0.93) years. As BLLs were not normally distributed, these were reported as median and interquartile range (IQR). In the overall sample, the median (IQR) BLL was 5.1 (3.6–7.1) μg/dL. The median (IQR) BLL was significantly higher in females [5.4 (3.3–7.6) μg/dL] than in males [4.9 (3.8–6.5) μg/dL; *p* = 0.001] (Table 1). In the overall sample, 16 students (5 male and 11 female) had BLLs > 30 μg/dL. Table 2 shows the distribution of students in various categories of BLLs. Approximately 3% of students had BLLs > 20 μg/dL. The distribution of BLLs in students from different Governorates are shown in Fig. 2A and with stratification by sex in Fig. 2B. BLLs were significantly higher in the Al-Asima, Mubarak Al-Kabeer and Al-Ahmadi Governorates, as compared to the other three Governorates (*p* < 0.001). Significant sex-Governorate interaction was observed (p < 0.001). As shown, the entire distributions of male students in Al-Asima and of female students in Mubarak Al-Kabeer and Al-Ahmadi Governorates were above the CDC reference BLL level (≥5 μg/dL). The distributions of BLLs across the age group categories (Table 1) were not significantly different (*p* = 0.07). However, age-Governorate interactions were significant (*p* < 0.001; Fig. 2C).

**Table 1:** Distribution of BLLs in adolescents based on demographic and nutritional variables

		N	Median (IQR)	Range Low - High	^a^High extreme values	***P***-value
	**Overall Sample**	1388	5.10 (3.60–7.13)	1.31–75.67	58.14–75.67	
**Sex**	Male	674	4.87 (3.78–6.49)	1.31–52.87	30.81–52.86	0.38
	Female	714	5.42 (3.25–7.58)	1.71–75.67	58.14–75.67	
**Nationality**	Kuwaiti	1064	4.9 (3.36–6.93)	1.31–75.67	58.14–75.67	< 0.001
	Non-Kuwaiti	324	5.28 (4.31–7.64)	1.93–45.6	24.41–45.6	
**Age**	10 to < 12 years	499	5.29 (3.74–7.36)	2.10–75.67	56.61–75.67	0.07
	12 to < 13 years	428	4.94 (3.55–6.67)	1.31–29.30	24.41–29.30	
	13+ years	434	4.97 (3.55–7.58)	1.73–72.57	40.6–72.57	
**BMI categories**	Normal weight	606	5.27 (3.69–7.20)	1.71–75.67	30.81–75.67	0.16
	Overweight	314	4.98 (3.62–6.81)	1.89–72.57	34.81–72.57	
	Obese	468	4.96 (3.48–71.30)	1.31–67.23	40.6–67.23	
**Anemia status**	Anemic	108	5.41 (3.50–8.25)	1.91–28.02	16.08–28.02	0.18
	Non-anemic	1253	5.05 (3.60–7.07)	1.31–75.67	58.14–75.67	
**Passive smoking**	Yes	478	5.00 (3.55–7.14)	1.77–75.67	28.59–75.67	0.81
	No	883	5.11 (3.60–7.08)	1.31–72.57	45.46–72.57	

*P*-values are based on non-parametric (Mann-Whitney or Kruskall-Wallis) tests comparing the distribution of of BLLs between groups within each demographic variable

*BLL* blood lead level, *IRQ* interquartile range; ^a^range of 5 high extreme values

**Table 2:** Distribution of BLLs based on different cutoff points in various demographic groups

			BLL cutoff (μg/dL)				
		*N*	< 5 *N* (%)	≥ 5 to < 10 *N* (%)	≥ 10 to ≤ 20 *N* (%)	> 20 *N* (%)	*P*-value
**Overall Sample**		1388	672 (48.4)	537 (38.7)	142 (10.2)	37 (2.7)	
**Governorate**	Hawally	231	207 (89.6)	14 (6.1)	7 (3.0)	3 (1.3)	< 0.001
	Farwaniya	230	178 (77.4)	43 (18.7)	5 (2.2)	4 (1.7)	
	Al-Jahra	240	108 (45.0)	115 (47.9)	16 (6.7)	1 (.4)	
	Al-Asima	187	40 (21.4)	95 (50.8)	49 (26.2)	3 (1.6)	
	Al-Ahmadi	350	92 (26.3)	203 (58.0)	43 (12.3)	12 (3.4)	
	Mubarak Al-Kabeer	147	47 (32.0)	64 (43.5)	22 (15.0)	14 (9.5)	
**Sex**	Male	674	357 (53.0)	232 (34.4)	71 (10.5)	14 (2.1)	0.004
	Female	714	315 (44.1)	305 (42.7)	71 (9.9)	23 (3.2)	
**Nationality**	Kuwaiti	1064	532 (50.0)	401 (37.7)	104 (9.8)	27 (2.5)	0.19
	Non-Kuwaiti	324	140 (43.2)	136 (42.0)	38 (11.7)	10 (3.1)	
**Age**	10 to < 12 years	499	229 (44.5)	209 (40.7)	60 (11.7)	16 (3.1)	0.008
	12 to < 13 years	428	222 (51.6)	174 (40.5)	27 (6.3)	7 (1.6)	
	13+ years	434	221 (49.8)	154 (34.7)	55 (12.4)	14 (3.2)	
**Weight status**	Normal weight	606	279 (46.0)	238 (39.3)	73 (12.0)	16 (2.6)	0.56
	Overweight	314	158 (50.3)	119 (37.9)	28 (8.9)	9 (2.9)	
	Obese	468	235 (50.3)	180 (38.5)	41 (8.8)	12 (2.6)	
**Anemia status**	Anemic	108	47 (42.3)	46 (41.4)	16 (14.4)	2 (1.8)	0.31
	Non-anemic	1253	625 (49.0)	490 (38.4)	126 (9.9)	35 (2.7)	
**Passive smoking**	Yes	478	235 (49.0)	188 (39.2)	49 (10.2)	8 (1.7)	0.38
	No	883	424 (47.9)	341 (38.5)	91 (10.3)	29 (3.3)	

*BLL* blood lead level. *P*-values are based on χ^2^ test comparing the distribution of students in various cutoffs of BLL in each category

The proportion of students with various BLLs in different demographic groups are shown in Table 2. Of the total sample, 713 (51.5%) had BLLs ≥5 μg/dL (47.0% of males and 55.8% of females; χ^2^ = 10.87; p < 0.001). In the Governorates of Al-Jahra, Al-Ahmadi, Al-Asima and Mubarak Al-Kabeer, the proportion of students with BLLs ≥5 μg/dL ranged from 55 to 79%, and the proportion of students with BLLs ≥10 μg/dL in Al-Ahmadi, Al-Asima and Mubarak Al-Kabeer ranged from 16 to 28% (Table 3). Similar to Fig. 2B, significant sex-Governorate interactions were noted in the proportions of students with EBLLs at both cutoffs (Table 3). Of the total population, 12.9% had BLLs of ≥10 μg/dL. Table 4 shows the association of various demographic variables with EBLLs. In the adjusted binary logistic regression, the female sex had significantly increased odds of having EBLL [OR (95%CI = 1.52 (1.22, 1.88)]. On the other hand, age between 12 to < 13 years had significantly lower odds of having EBLLs [OR (95%CI = 0.75 (0.58, 0.97)] compared to the other age groups. Compared to the Governorate of Hawally (the reference Governorate), the OR of having EBLLs in other Governorates were significantly higher (Table 4). Nationality, weight status, anemia status and passive smoking did not show association with EBLL.

**Table 3:** Percentages of students with EBLLs in all Governorates based on two cutoff points of BLL

	BLL ≥5 μg/dL^a^			BLL ≥10 μg/dL		
	Overall sample	Male	Female	Overall sample	Male	Female
Hawally	10.4 (231)	13.0 (123)	7.4 (108)	4.3 (231)	6.5 (123)	1.9 (108)
Farawanya	22.6 (230)	34.1 (82)	16.2 (148)	3.9 (230)	2.4 (82)	4.7 (148)
Al-Jahra	55.0 (240)	47.5 (118)	62.3 (122)	7.1 (240)	11.9 (118)	2.5 (122)
Al-Asima	78.6 (187)	92.9 (98)	62.9 (89)	27.8 (187)	20.4 (98)	36.0 (89)
Al-Ahmadi	73.7 (350)	53.7 (177)	94.2 (173)	15.7 (350)	19.2 (177)	12.1 (173)
Mubarak Al-Kabeer	68.0 (147)	40.0 (75)	97.2 (72)	24.5 (147)	9.3 (75)	40.3 (72)
χ^2^	374.6	149.9	354.1	95.7	25.0	120.1
p-value	< 0.001	< 0.001	< 0.001	< 0.001	< 0.001	< 0.001

The numbers in parenthesis in each cell are the number of students (total, male or female) in each Governorate that were used as denominator to calculate the percentages

^a^Includes all students with BLL ≥5 μg/dL (inclusive of BLL ≥10 μg/dL category)

**Table 4:** Odds ratio of EBLL (BLL ≥5 μg/dL) associated with demographic and nutritional variables

Demographic Variables		UOR	95% CI	AOR	95% CI
**Governorate**	Hawally	1.00	Ref	1.00	Ref
	Farwaniya	2.65	1.57, 4.49	2.55	1.49, 4.35
	Al-Jahra	9.88	6.01, 16.23	11.16	6.58, 18.94
	Al-Asima	32.30	18.57, 56.20	34.39	19.56, 60.45
	Al-Ahmadi	24.69	15.18, 40.17	29.63	17.53, 50.08
	Mubarak Al-Kabeer	18.29	10.56, 31.66	19.21	10.98, 33.58
**Sex**	Male	1.00	Ref	1.00	Ref
	Female	1.52	1.22, 1.88	1.89	1.40, 2.54
**Age**	10 to < 12 years	1.00	Ref	1.00	Ref
	12 to < 13 years	0.75	0.58, 0.97	0.70	0.51, 0.95
	13 or above	0.80	0.61, 1.03	0.87	0.63, 1.19
**Nationality**	Kuwait	1.00	Ref	1.00	Ref
	Non-Kuwaiti	0.78	0.60, 1.00	0.82	0.56, 1.19
**Weight status**	Normal weight	1.00	Ref	1.00	Ref
	Overweight	0.88	0.67, 1.17	0.99	0.71, 1.38
	Obese	0.84	0.66, 1.07	1.02	0.76, 1.38
**Anemia status**	No	1.00	Ref	1.00	Ref
	Yes	1.24	0.83, 1.85	0.82	0.51, 1.31
**Passive smoking**	No	1.00	Ref	1.00	Ref
	Yes	0.96	0.76, 1.20	0.99	0.71, 1.38

Odds ratios were calculated using binary logistic regression. BLL were categorized as < 5 μg/dL (reference) or ≥ 5 μg/dL (EBLL). In the adjusted model, all the variables in the first column were included

*UOR* unadjusted odds ratio, *AOR* adjusted odds ratio

## Discussion

This study revealed that over 51% of the adolescent population in Kuwait had BLLs above the CDC reference level (≥5 μg/dL) with 13% of the study population having BLLs of > 10 μg/dL). The Governorates of Al-Asima, Al-Ahmadi and Mubarak Al-Kabeer stood out as areas with the highest BLLs. PB poisoning is one of the major public health concerns globally with developing nations hit hard because of socio-cultural and environmental factors along with a lack of firm regulations against the use of Pb in commodities [3]. There is no known safe level of exposure, particularly in children. Since the revision of the BLL reference level in 2012, there is a great need to reassess the population Pb exposure, particularly in areas where the average exposure level was ≥5 μg/dL. The presence of heavy petrochemical industry, and the heavy traffic burden makes Kuwait a high Pb exposure environment [12]. In addition, the excessive use of ammunition and the burning of oil wells during the 1991 Gulf War might also have had its impact on environmental Pb levels. However, studies on the population Pb exposure, using representative samples, are non-existing. Studying Pb exposure in children and adolescents is particularly important due to the well-known and long-lasting adverse effects of low-level Pb exposure on brain development and function [24]. Approximately, 25% of the population in Kuwait is under the age of 15 years [25]. This study is based on a nationally representative sample of adolescent school children.

The median BLLs was 5.1 μg/dL. However, a significant proportion of students (51%) had BLL above the CDC/WHO cutoff of ≥5 μg/dL, and this proportion was even higher (56%) in female students. About 13% of the students had BLLs ≥10 μg/dL. Similar findings have been reported in children and adolescents (age range 4–19 years) from Saudi Arabia [mean BLL 4.9 μg/dL; 50% with BLLs ≥5 μg/dL] [26], Indonesia [47% with BLLs ≥5 μg/dL] [27], and Egypt [mean BLL 5.6 μg/dL; 57% with BLLs ≥10 μg/dL] [28]. The prevalence of EBLLs in our study was lower than many developing countries such as Senegal [61.7% > 10 μg/dL] [29], Bangladesh [87.4% > 10 μg/dL] [30], Nepal [84% > 10 μg/dL] [31], India [47% > 10 μg/dL] [32], Mexico [64% > 5 μg/dL] [33] and south African countries [80–98% > 10 μg/dL] [34]. On the other hand, the average BLLs from many developed countries were lower than the median BLL in this study. Examples are average BLLs from the United States [1.9 μg/dL; 3.8% > 5 μg/dL] [35], Sweden [1.4 μg/dL], Poland [1.63 μg/dL], Czechia [1.55 μg/dL], Slovakia [1.9 μg/dL] [36], France [1.49 μg/dL] [37], and Canada [0.48 μg/dL] [38]. Possible reasons for these variations in the prevalence of EBLLs include variations in the study populations (whether from general populations or high exposure environments), age, and the degree of implementing legal restrictions to minimize or restrict the use of Pb [39, 40].

Striking differences were observed in Pb exposure among the six Governorates, both in the median BLLs and in the proportions of students with EBLLs. These differences remained significant after adjusting for the various known factors associated with EBLL such as age, sex, smoking, and anemia. In terms of the median BLL, the entire distribution of male students in Al-Asima and female students in Mubarak Al-Kabeer and Al-Ahmadi were above the CDC reference level of ≥5 μg/dL (Fig. 2B). The Governorates of Al-Asima, Al-Ahmadi (both males and females), Al-Jahra (males) and Mubarak Al-Kabeer (females) stood out as the areas with the highest proportions of students with EBLLs, particularly above the cutoff of ≥10 μg/dL (Table 3). Two previous studies [41, 42] from Kuwait also have reported significant differences between the residents from residential and industrial areas.

Several factors could be responsible for the EBLLs in these areas. Major potential sources in Al-Asima Governorate include the presence of major highways with heavy traffic burden, the presence of old buildings, the main commercial shipping port, hundreds of automobile repair workshops, and an industrial area. Potential sources in Al-Ahmadi Governorate include the presence of oil fields and refineries. In addition, this Governorate has an agricultural site (Al-Wafra), where the use of pesticides and fertilizers could contribute to the soil and air Pb content. Soil and air samples from these two Governorates have been reported to contain higher levels of Pb [13, 15, 43, 44]. The Governorate of Mubarak Al-Kabeer is located next to the Al-Ahmadi Governorate and the spill-over of the environmental Pb is suspected to be the reason for EBLLs. Being a newly developed area with a lot of construction activity, the exhaust from the construction machinery and the rising dust might also contribute to Pb exposure. The particularly high Pb exposure in the female school from this Governorate is of significant public health concern as there might be a local source of contamination. The unsuitability of drinking water due to the lack of maintenance of filters, internal water supply networks, reservoirs and chillers for drinking water in some of the schools from this Governorate has been reported in 2018 (Al-Anba Newspaper dated March 2, 2018). Similarly, the male school in the Al-Jahra Governorate is located in close proximity to two major industrial zones (Jahra and Sulaibiya), a waste-water treatment plant, an electrical cable and appliances factory, an oil and pipe manufacturing unit, and a lead-acid batteries (LAB) recycling unit. LAB manufacturing and recycling factories are still considered a source of Pb contamination of the soil, air and water in developing countries [27, 39], as well as in developed countries despite all the regulations and monitoring by the EPA [45]. Although the Kuwait EPA has several regulations (Law Number 42; Decision No. 8, for example) to monitor and restrict the emission of toxic wastes, it is not clear to what degree these restrictions are implemented. The Governorates of Hawally and Farwaniya are mostly residential and, although very congested with a heavy traffic burden, had lower BLL. This suggests that the source of Pb exposure in Kuwait is mostly from oil installations and industrial activity.

Flaring of the unwanted gases from extracted crude oil is considered as a major source of emission and air pollution in oil producing countries [46]. Heavy metals including cadmium (Cd), mercury (Hg) and Pb are found in crude oil and gas and are emitted to the environment from flaring [47]. The Middle East has the highest flaring rate with approximately 81 metric tons (MT) in the year 2018 [48]. The emission of Pb from flaring is estimated to be 4.3 mg Pb/ton throughput from the associated gases and 5.4 mg/ton throughput from the non-associated gases [49]. Thus, in Kuwait, emission of Pb into the environment from gas flaring is a potential source of environmental Pb. This may be particularly relevant in the Al-Ahmadi and Mubarak Al-Kabeer Governorates, which are close to oil installations. The Kuwait National Petroleum Company (KNPC) has adopted several measures to curb emissions and to protect the nearby communities [50]. In 2018, it installed a fully functional flare gas recovery unit to curb emissions. However, the Pb emitted from gas flaring in the past may still exist in the environment (air and soil), which may still be a source of environmental exposure.

The median BLL was significantly higher in female students compared to male students, however, this relationship was not the same across all the Governorates. In some Governorates, males had significantly higher BLL than females and the opposite was true in others; and no difference in still others. A similar pattern was also observed in the proportion of students with EBLLs either at ≥5 μg/dL or the ≥10 μg/dL cutoff points. In this study, female sex was significantly associated with EBLLs. The association of sex with EBLLs is not consistent across studies. Some studies reported no association between EBLL and sex [28, 35] while others have reported significant differences between males and females in the same age group [26, 36, 51]. Higher prevalence of anemia, and the use of “kohl” and other cosmetics, that may be contaminated with Pb, could contribute to EBLL in girls compared to boys [52–54]. Anemia is this study population was 11% in females and 5% in males [55].

To our knowledge, this is the first properly-designed study utilizing a nationally representative sample of adolescents from Kuwait. We employed a very sensitive method of Pb estimation (ICP-MS) with strict quality control measures. Our method recovered 96.8% of Pb in Standard-1 (S1) and 96% in S2 (Seronorm samples with known Pb content). There are, however, a few limitations in this study. First, dietary history and food intake was not evaluated as possible factors of Pb exposure. Second, parental occupational details were also not taken into account which could provide a clue to the exposure source. Third, students were selected from schools and Pb data from other members of the household was not available. As such, it is difficult to discern whether the source of Pb is from households or from the schools.

## Conclusions

More than half of the adolescent population in Kuwait had BLLs above the CDC reference level (≥5 μg/dL), and almost 13% had BLLs ≥10 μg/dL, suggesting that EBLL is a public health concern. Furthermore, BLLs were particularly high in some Governorates like Al-Asima, Mubarak Al-Kabeer and Al-Ahmadi. More in-depth studies are needed to identify the exact source of exposure in these areas. Re-evaluating Pb toxicity in children with a wider age range is warranted. Public health interventions are urgently required to reduce the exposure in these areas to protect children from the harmful effects of Pb toxicity. In addition, public awareness campaigns should be launched in schools as a preventive measure to educate children, teachers and parents about Pb poisoning and its exposure risk factors.

## Supplementary Information

**Additional file 1: Data Acquisition Tool.** English translation of a self-administered questionnaire for parents and structured questionnaire for students for a face-to-face interview. Original questionnaires were developed and administered in Arabic.

## Data Availability

The datasets used and/or analyzed during the current study are available from the corresponding author on reasonable request.

## Abbreviations

BLL: Blood lead level
EBLL: Elevated blood lead level
EPA: Environment public authority
ICP-MS: Inductively coupled plasma mass spectrometry
IRQ: Inter-quartile range
LAB: Lead-acid batteries
MT: Metric ton
zBMI: BMI-for-age z-score
