# Supplementary material for: Prevalence of lead toxicity in adolescents in Kuwait
Source: BMC Public Health. 2021 Jun 22;21:1189. doi: 10.1186/s12889-021-11210-z (PMC8220793; doi:10.1186/s12889-021-11210-z)
Supplement: Supplementary file 1 — Additional file 1: Data Acquisition Tool. English translation of a self-administered questionnaire for parents and structured questionnaire for students for a face-to-face interview. Original questionnaires were developed and administered in Arabic. [file 12889_2021_11210_MOESM1_ESM.pdf]

	Study ID number	[________]

Association of Vitamin D Levels with Cognitive Function and School Performance in School Children in Kuwait.

Parents' Form

	Study ID number	[_________]

No	Question	Categories	Code
**Q1**	What is your relation to the student who is participating in the study?	Father(1)<br>Mother(2)<br>Other relative(3) (specify):_________________	
**Q2**	In which governorate do you currently live?	Capital(1)<br>Hawally(2)<br>Farawanya(3)<br>Jahra(4)<br>Mubarak Al-Kabeer(5)<br>Ahmadi(6)	
**Q3**	What is the father's highest level of education completed?	No formal education(1)<br>Completed primary school(2)<br>Completed secondary school(3)<br>Completed university(4)<br>Higher than university degree(5)	
**Q4**	What is the mother's highest level of education completed?	No formal education(1)<br>Completed primary school(2)<br>Completed secondary school(3)<br>Completed university(4)<br>Higher than university degree (5)	
**Q5**	What is the father's current income per month?	Less than 500 KD(1)<br>500 – 1000 KD(2)<br>1501 – 2000 KD(3)<br>More than 2000 KD(4)<br>Don't know(5)	
**Q6**	What is the current employment status of the mother?	Housewife(1) → skip to Q8<br>Paid employed(2)<br>Other(3) (specify):_________________	
**Q7**	What is the mother's current income per month?	Less than 500 KD(1)<br>500 – 1000 KD(2)<br>1501 – 2000 KD(3)<br>More than 2000 KD(4)<br>Don't know(5)	

	Study ID number	[________]

No	Question	Categories	Code
**Q8**	Do you currently live in:	Rented flat?(1)<br><br>Rented house?(2)<br><br>Owned flat?(3)<br><br>Owned house?(4)	
**Q9**	How many brothers and sisters does your son/daughter (who is participating in the study) have currently?	Brother:______________<br><br>Sisters:________________	|__|__|
**Q10**	Does your son/daughter (who is participating in the study) have his/her own room?	Yes(1)<br><br>No(2)	
**Q11**	What is the birth order of your son/daughter (who is participating in the study) from his/her mother?	First child (1)<br><br>Second child (2)<br><br>Third child (3)<br><br>Fourth child (4)<br><br>Fifth child or more(5)	
**Q12**	Does anyone smokes cigarettes inside your home?<br>(Don't consider those who smoke cigarettes only outside your home)	No(0)<br><br>Yes(1) (who):___________________<br>e.g. father, mother, brother	
**Q13**	Does anyone smokes Shisha inside your home?<br>(Don't consider those who smoke Shisha only outside your home)	No(0)<br><br>Yes(1) (who):___________________<br>e.g. father, mother, brother	
**Q14**	On a typical week, how often does your child have a meal before going to the school?	Every day/5 days per week (1)<br><br>4 days per week(2)<br><br>3 days per week(3)<br><br>2 days per week(4)<br><br>1 days per week(5)<br><br>Never(6)	|___|

Q15. During the past 3 months, how many times each week (including weekdays and weekends) has your son/daughter (who participated in the study) eaten breakfast not prepared at home?

- ☐ 0 Never or almost never
- ☐ 1 1-2 times per week
- ☐ 2 3-4 times per week
- ☐ 3 5 or more times per week

	Study ID number	[________]

Q16. During the past 3 months, how many times each week (including weekdays and weekends) has your son/daughter (who participated in the study) eaten lunch <u>not prepared at home</u>?

- ☐ 0 Never or almost never
- ☐ 1 1-2 times per week
- ☐ 2 3-4 times per week
- ☐ 3 5 or more times per week

Q17. During the past 3 months, how many times each week (including weekdays and weekends) has your son/daughter (who participated in the study) eaten dinner <u>not prepared at home</u>?

- ☐ 0 Never or almost never
- ☐ 1 1-2 times per week
- ☐ 2 3-4 times per week
- ☐ 3 5 or more times per week

	Study ID number	[_________]

Association of Vitamin D Levels with Cognitive Function and School Performance in School Children in Kuwait.

Student's Form

**Today date:**	-----/-----/----------
**Child's name:**	: --
**Civil ID:**	:-------------------------
**School's name:**	: -- **Code:**-------
**Telephone number:**	**Home number:**-------------------------------------<br>**Mother's mobile:**-----------------------------------<br>**Father's mobile:**------------------------------------
**School's Address:**	: --<br>--<br>---<br>---<br>--
**School's Governorate:**	☐ Capital(1) ☐ Hawally(2) ☐ Farawanya(3)<br>☐ Jahra(4) ☐ Mubarak Al-Kabeer(5) ☐ Ahmadi(6)
**Number of the students in the school**	
**Study ID number:**	---

	Study ID number	[_________]

No	Question	Categories	Code
**Q1**	Gender	□Male(1)<br><br>□Female(2)	
**Q2**	What is your current nationality?	□Kuwaiti(1)<br><br>□Non-Kuwaiti Arab(2) (specify):___________<br><br>□Non-Kuwaiti non-Arab(3) (specify):________	
**Q3**	What is your Date of Birth?	DD MM YY<br>______/_______/________/	
**Q4**	Which grade are you currently in?	_____________e.g. sixth, seventh	
**Q5**	Who do you currently live with?	□ Father and mother(1)<br><br>□ Mother but not with the father(2)<br><br>□ Father but not with the mother(3)<br><br>□ Other relatives but not with the father or mother(4)	
**Q6**	Does your father, mother or any family member helps you in your study? E.g. doing homework, understanding difficult subjects.	□Yes, regularly (1)<br><br>□Yes, sometimes (2)<br><br>□Yes, rarely (3)<br><br>□No (4)	
**Q7**	Do you have personal tutor to help you in your study?	□No(0)<br><br>□Yes(1) (In which subjects):________________<br>e.g. Math, Science, Arabic literature..etc	
**Q8**	Do you currently use any medications or supplements like vitamins?	□No(0)<br><br>□Yes(1) (specify):__________________	
**Q9**	Does anyone smoke cigarettes inside your home?<br>(Don't consider those who smoke cigarettes only outside your home)	□No(0)<br><br>□Yes(1) (who?):___________________<br>e.g. father, mother, brother	
**Q10**	Does anyone smoke Shisha inside your home?<br>(Don't consider those who smoke Shisha only outside your home)	□No(0)<br><br>□Yes(1) (who?):___________________<br>e.g. father, mother, brother	
**Q11**	Do you currently smoke cigarettes?	□No(0)<br><br>□Yes(1)(How many cigarettes per day?):_______	
**Q12**	Do you currently smoke Shisha?	□No(0)<br><br>□Yes(1) (How many times per week?):_______	
**Q13**	In a typical weekday (Sunday-Thursday), how many hours do you sleep during the night?	Probe by asking about the time going to bed, falling asleep and awakening in the morning and going school. _______hours	
**Q14**	In a typical weekday (Sunday-Thursday), do you sleep during day-time?	□No(0)<br><br>□Yes(1) (How many hours per day?):_______	

	Study ID number	[________]

No	Question	Categories	Code
**Q15**	In a typical weekend (Friday-Saturday), how many hours do you sleep during the night?	Probe by asking about the time going to bed, falling asleep and awakening in the morning and having breakfast or going out. _______hours	
**Q16**	In a typical weekend (Friday-Saturday), do you sleep during day-time?	No(0)<br>Yes (1) (On average how many hours?):_______	
**Q17**	Do you have any health condition that limits your physical activity?	No(0)<br>Yes(1) (specify):__________________	
**Q18**	In school, how many times per week do you attend physical education classes?	None(0)<br>Once(1)<br>Twice(2)<br>Three Times(3)<br>Four Times(4)<br>Five Times or more(5)	
**Q19**	During PAST WEEK, how many times did you walk to the school?<br>(Going and coming from school=2 times)	_____________Times<br>If none, put zero.	
**Q20**	If you walked to the school, how many minutes you walk to reach the school?	______________Minutes	
**Q21**	During PAST WEEK, how many times did you travel to the school by car or bus?	________ Times	
**Q22**	Over the past 3-months, on average, how many hours are you outside per day between 10:00 am and 4:00 pm **on Weekdays (Sunday to Thursday)**?	less than 30 minutes(0)<br>31 minutes to 1 hour(1)<br>More than 1 hour to 2 hours (2)<br>More than 2 hours to 3 hours(3)<br>More than 3 hours to 4 hours (4)<br>More than 4 hours (5)	
**Q23**	Over the past 3-months, on average, how many hours are you outside per day between 10:00 am and 4:00 pm **on Weekend days (Friday and Saturday)**?	less than 30 minutes(0)<br>31 minutes to 1 hour(1)<br>More than 1 hour to 2 hours (2)<br>More than 2 hours to 3 hours(3)<br>More than 3 hours to 4 hours (4)<br>More than 4 hours (5)	
**Q24**	In the past 12-months, how many times did you have a red or painful sunburn that lasted a day or more?	None(0)<br>One (1)<br>Two (2)<br>Three(3)<br>Four (4)<br>Five and more (5)	

	Study ID number	[_________]

Q25. For the following questions, think about what you do when you are outside during the past 3-months?

	Never(0)	Rarely(1)	Sometimes(2)	Often(3)	Always(4)
a. How often do you wear sunscreen?	☐	☐	☐	☐	☐
b. How often do you wear a shirt with sleeves that cover your shoulder?	☐	☐	☐	☐	☐
c. How often do you wear a hat?	☐	☐	☐	☐	☐
d. How often do you stay in the shade or under an umbrella?	☐	☐	☐	☐	☐
e. How often do you spend time in the sun in order to get a tan?	☐	☐	☐	☐	☐

No	Question	Categories	Code
**Q26**	On a typical **Weekday (Sunday-Thursday)** at what time do you have your first meal?	☐ Before 7 am (0)<br>☐ 7-8 am (1)<br>☐ 8-9 am (2)<br>☐ 9-10 am (3)<br>☐ After 10 am (4)	
**Q27**	On a typical **Weekend (Friday and Saturday)** at what time do you have your first meal?	☐ Before 7 am (0)<br>☐ 7-8 am (1)<br>☐ 8-9 am (2)<br>☐ 9-10 am (3)<br>☐ After 10 am (4)	
**Q28**	How many times **per week** do you drink sugary drink/soft drinks (e.g. Coke, Pepsi, 7up, Sports drinks)?	☐ None(0)<br>☐ Once (1)<br>☐ Twice(2)<br>☐ 3 times (3)<br>☐ 4 times (4)<br>☐ 5 times (5)<br>☐ 6 times (6)<br>☐ 7 times or more (7)	

	**Study ID number**	**[________]**

QUESTIONS 29- 32 for female students ONLY.

Q29 Have you started your period yet?

☐1 Yes → answer questions 30-32

☐2 No → skip to food frequency questionnaire

If yes:

Q30 What was your age when your first period began? _________years _________months

Q31 What was the date of your first period? _______year _______month _______day

Q32 In which school grade did your first period begin? ______

Q33) Choose the most picture that match the student's dress today.

1

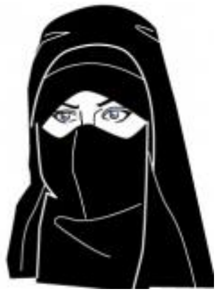

2

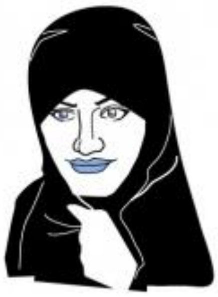

3

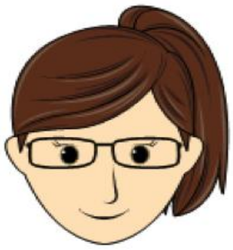

	Study ID number	[_________]

Anthropometric Measurements

**Weight (Kgs):**	___________kg
**Height (Cms):**	___________cm
**Waist (Cms):**	___________cm

The data below should be extracted from school's record:

Student's ID : [ ]

**Student's Marks:**	**Mathematics**	**Science**	**Arabic Language**	**Total Mark**
